# DNA bending in the synaptic complex in V(D)J recombination: turning an ancestral transpososome upside down

**DOI:** 10.15190/d.2014.5

**Published:** 2014-03-29

**Authors:** Mihai Ciubotaru, Marius Surleac, Mihaela G. Musat, Andreea M. Rusu, Elena Ionita, Paul C. C. Albu

**Affiliations:** Department of Immunobiology, Yale University School of Medicine, 300 Cedar St., TAC S620, New Haven, CT 06511, USA; National Institute for Physics and Nuclear Engineering Horia Hulubei, Department of Life and Environmental Physics, Atomistilor Str., 077125, Bucharest-Magurele, Romania; Department of Bioinformatics and Structural Biochemistry, Institute of Biochemistry of the Romanian Academy, Splaiul Independentei 296, 060031, Bucharest, Romania

**Keywords:** VDJ recombination, RAG, RAG1, phosphoryl transfer reaction, recombination versus transposition, transposon, mobile elements in human genome

## Abstract

In all jawed vertebrates RAG (recombination activating gene) recombinase orchestrates V(D)J recombination in B and T lymphocyte precursors, assembling the V, D and J germline gene segments into continuous functional entities which encode the variable regions of their immune receptors. V(D)J recombination is the process by which most of the diversity of our specific immune receptors is acquired and is thought to have originated by domestication of a transposon in the genome of a vertebrate.  RAG acts similarly to the cut and paste transposases, by first binding two recombination signal DNA sequences (RSSs), which flank the two coding genes to be adjoined, in a process called synaptic or paired complex (PC) formation. At these RSS-coding borders, RAG first nicks one DNA strand, then creates hairpins, thus cleaving the duplex DNA at both RSSs. Although RAG reaction mechanism resembles that of insect mobile element transposases and RAG itself can inefficiently perform intramolecular and intermolecular integration into the target DNA, inside the nuclei of the developing lymphocytes transposition is extremely rare and is kept under proper surveillance. Our review may help understand how RAG synaptic complex organization prevents deleterious transposition. The phosphoryl transfer reaction mechanism of RNAseH-like fold DDE motif enzymes, including RAG, is discussed accentuating the peculiarities described for various transposases from the light of their available high resolution structures (Tn5, Mu, Mos1 and Hermes). Contrasting the structural 3D organization of DNA in these transpososomes with that of the RSSs-DNA in RAG PC allows us to propose several clues for how evolutionarily RAG may have become “specialized” in recombination versus transposition.

## SUMMARY

1. Introduction
2. V(D)J recombination outline, RAG proteins
3. RAG1 similarities with other transposases
  - 3.1. Specific DNA Binding Domain (DBD)
  - 3.2. The Catalytic Domain (CD)
  - 3.3. Nonspecific/Target DNA Binding Domain (TB)
4. RAG DNA cleavage mechanism; a comparative view with other transposase phosphoryl transfer reactions
5. Paired complex organization and RSS bending in V(D)J recombination versus transpososome /intasome architecture
6. Recombination versus transposition: when the ends meet
7. Conclusions

## 1. Introduction

**Figure 1:**
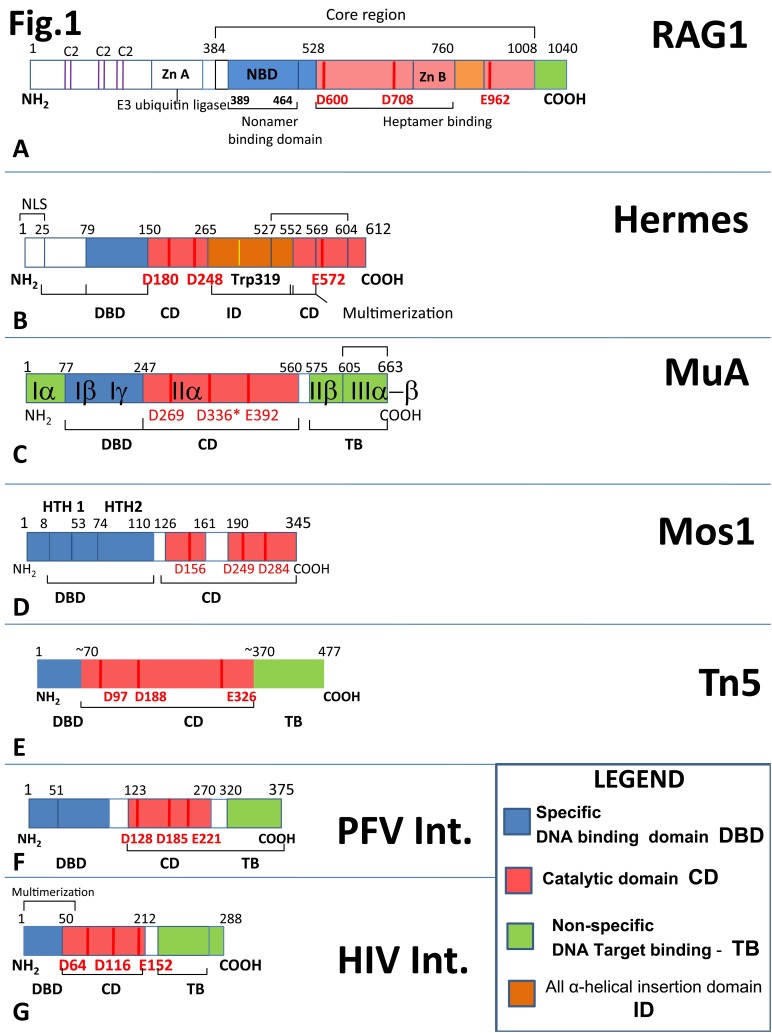
Linear alignment of the transposase/ integrase family proteins Linear alignmentdescribed in this comparative study is displaying in colors their main common trimodular features. The specific DNA binding domains in Blue(DBD), the catalytic domain (CD) in Red with the triad DDE/D motif shown as red bars, the nonspecific target DNA binding sites (TB) in green. The diagrams also point the CD all α helical insertion domains in orange(ID). **A.** RAG1, **B.** Hermes, **C.** MuA, **D.** Mos1, **E.** Tn5, **F.** PFV-Int., **G.** HIV-Int.

**Figure 2:**
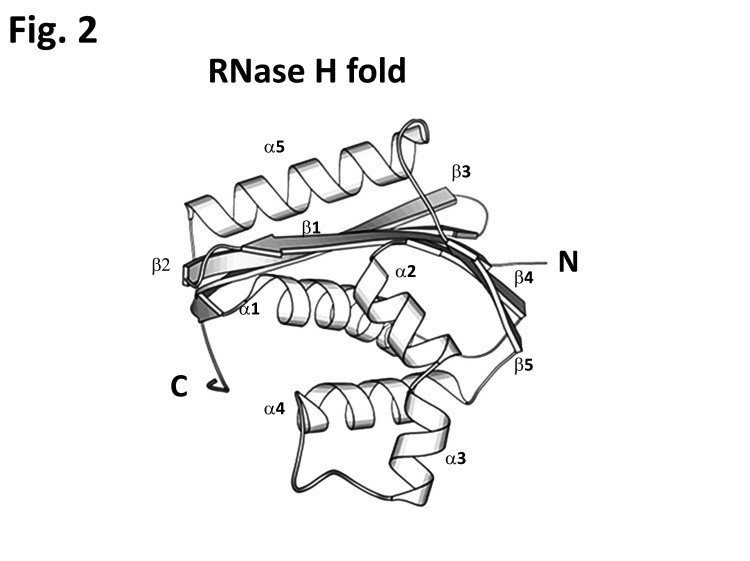
E. coli RNase H folding with a b1-b2-b3-a1-b4-a2/3-b5-a4-a5 sequence revealed in 1990 by Yang et al.^38^ (PDB code 1RNH.pdb).

Although in 2000 USA President Bill Clinton officially announced that a group of scientists from Celera lead by Greg Venter^1^ and Francis Collins from the National Institutes of Health U.S. Public Genome Project jointly mapped the human genome, the detailed sequenced project has been completely finalized only in 2003. Since then, and largely using Venter's fast shotgun sequencing approach, many genomes of organisms from the simplest bacteria to our cousins the primates have been completely read. Today's technology has tremendously improved and we now talk about moderate costs for sequencing individual human genomes. The take home message from this gigantic human endeavor revealed two striking features shared by all advanced eukaryotes. First, the number of genes encoding their functional proteins, denoted open reading frames (ORFs), is minute occupying only 2-3% of the genomic sequences. And if this were not surprising enough, later many sequenced genomes have revealed an even more intriguing fact, that sequences of transposomic origin abound in almost every living genome. Transposons, mobile elements or simply “selfish DNAs”, are sequences that mobilize from one place to another on the same or on another chromosome, from the same or different species. By far they represent the most abundant genes in any genome. To be active, the minimal requirement of their sequence is an active ORF encoding a transposase, which upon specific recognition of the two ends (denoted **I**nsertional **s**equences IS or **I**nverted **r**epeats IR) of the transposon, cuts them from its donor place, moving and inserting them to another acceptor or target DNA^2^. Such "hoping" activity can disrupt or mobilize genes around genome(s), hence, for long time it was thought that their arcane origin is just a reminder of our evolution, but they should be completely inactive, at least in evolved vertebrates. More than, 45% of the human genome is represented by such mobile elements and, even more surprising, there is strong evidence that LINE-1 (long interspersed nucleotide element-1, L1) retrotransposons, which comprise 17% of the human genome, are polymorphic and active in human populations^3^. Another family of transposons that do not use RNA but double-stranded DNA intermediates to spread, are the so called cut-and-paste DNA transposons. They are less abundant than retrotransposons in human genome but, unlike these, they are equally well spread in eukaryotes and prokaryotes. Strong evidence has accumulated in the last years to support the fact that around 450 million years ago, from an ancient DNA transposon, the vertebrates inherited and later evolved the enzyme, its reaction mechanism and the strategy to assemble the genes encoding the specific immunity effectors^4-8^. This process is called V(D)J recombination and provides the immune repertoire in all jawed vertebrates. There are five major arguments favoring this hypothesis all addressed in detail by our review: 1) RAG1 subunit of RAG recombinase, which recombines V,D,J segments, is highly homologous to many insect *Transib* transposases^9^; 2) RAG1 is a multimodular protein whose linear domain organization parallels that of many described transposases and viral DNA integrases^5,10,11^; 3) The specific RSS DNA elements recognized by RAG have similar consensus elements with those recognized and processed by *Transib* transposases TIR (*Transib* inverted repeats)^9^; 4) RAG catalytic mechanism of DNA cutting and hairpin end processing resembles that of insect transposases like *Hztransib* or *Hermes* with which RAG1 shares structural homology in its catalytic core domain^12,13^ (**Figure 1**, **Figure 2**); 5) With its cleaved end RSSs *in vitro* and *in vivo* RAG transposes their intervening DNA, but the process is inefficient and highly exceeded by the physiologic recombination reaction^4-7^.

We will contrast the details of yet poorly understood DNA-RAG complex molecular architecture with those of the known structures of four DNA transposases MuA, Mos1, Tn5, Hermes and of the Prototype Foamy Virus (PFV) Integrase^12,14-18^ in complexes with their donor and acceptor DNAs. From these structures, we will try to understand the underpinnings of RAG new function and how evolution has chiseled its way out from transposition into recombination. 

## 2. V(D)J recombination outline, RAG proteins

**Figure 3:**
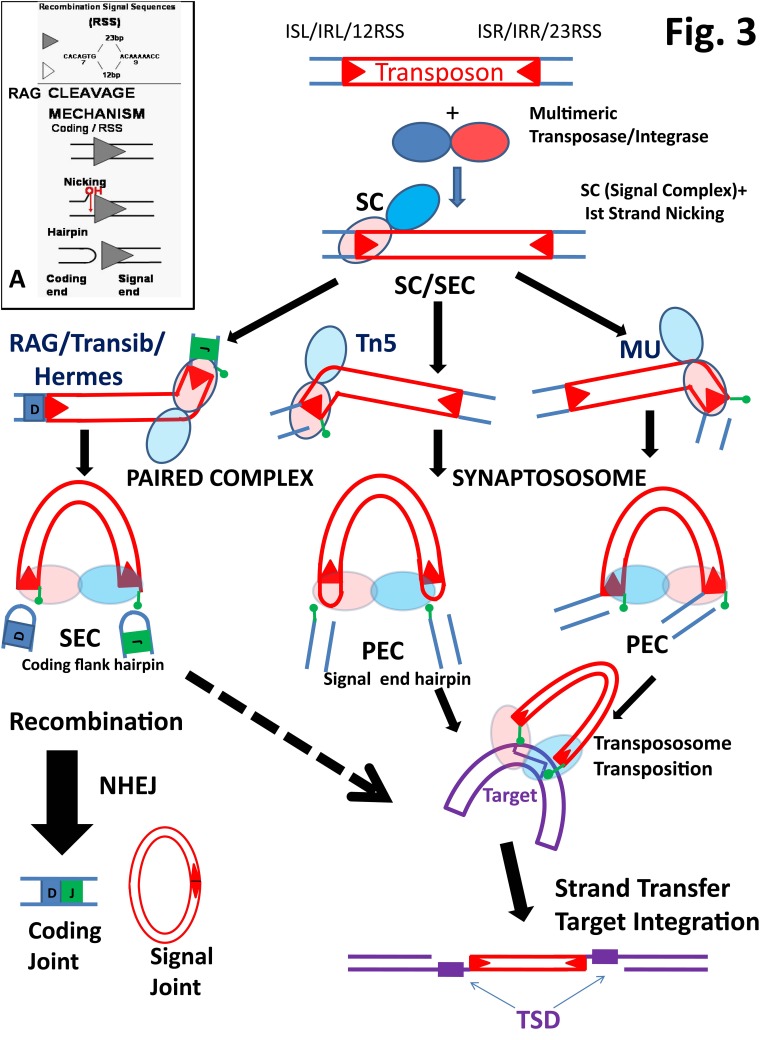
Schematic diagram of the mechanism of transposition and recombination reactions. Inset A, details of the RAG mechanism of cleavage are shown just at one RSS end. IS, IR- terminal Insertional sequences or inverted repeat terminal sequences of the mobile element. SC-signal complex. Green ovals denote the 3'OH groups freed by each of the two rounds of the nucleophilic attacks. PC-paired complex, PEC-paired end complex. SEC-signal end complex generated after two complete cuts are created and the element is disintegrated from the donor chromosome. NHEJ-nonhomologous end joining cellular enzymatic machinery involved in DNA repair. TSD- target site duplication.

In the nuclei of lymphoid cells RAG recombinase shuffles small germline gene segments called V (variable), D (diverse) and J (junctional), to assemble functional genes encoding the variable regions of their immune receptors (Immunoglobulins for B cells and T Cell Receptors TCRs for T lymphocytes)^10,19^. RAG first binds and then brings together a complementary (12/23) pair of DNA recombination sites - RSSs (**r**ecombination **s**ignal **s**equences), flanking the V,D,J coding genes to be adjoined, a process called paired complex (PC) or synapsis formation. The consensus RSSs encompass a short palindromic heptamer (7 base pairs- bp) and an A rich nonamer (9 bp), elements separated by a non-conserved spacer of 12 or 23 bp (**Figure 3**[A])^20,21^. *In vivo,* RAG recombinase creates double stranded breaks at the junction of the gene segments with their RSSs, only in the context of a 12/23-RSS synaptic or **p**aired **c**omplex (PC) (the 12/23-rule)^20^. Once the double stranded cuts are created, the RAG complex tightly holds the two signal ends in a **s**ignal **e**nd **co**mplex (SEC)^22^, which eventually generates a ligated signal joint (**Figure 3**). The coding hairpinned flanking ends are processed by the Nonhomologous End Joining Cellular Machinery generating the gene segment recombination product (see **Figure 3** and section 4 below).

RAG recombinase is made of two distinct polypeptidic subunits RAG1 and RAG2. In this review, we will describe the murine proteins, which were the most extensively studied RAG proteins since their initial discovery in 1989^23,24^. RAG1 is a 1040 aminoacids protein, highly homologous to many eukaryotic transposases^25-27^, or retroviral integrases like HIV Integrase (HIV-IN). RAG1 can be divided schematically in two regions (**Figure 1** [A]), the N terminus 1-383 aa. and the core domain 384-1040 (amino acids - aa.) of the C terminus. The N terminal domain contains three conserved pairs of Cysteine residues (C_2_ **Figure 1** [A]), a highly conserved zinc-binding motif A involved in dimerization and having an E3 ubiquitin ligase activity interacting with and ubiquitinating Histone H3^11^. RAG1 core, has a minimal region 384–1008 aa. supporting catalysis. Core RAG1, 377-1008 binds alone 12-RSS as a dimer in solution with a binding Kd ≈ 40nM, and upon doing so suffers a major conformational change^28^. RAG1 core has a short 389-464aa. nonamer binding domain (NBD) that confers specific nonamer RSS-DNA binding and dimerization properties as reflected in the solved high-resolution structure^29^. The aa. 528-760 central core domain of RAG1 has a second ZnB finger motif, which confers DNA heptamer recognition and perhaps RAG2 interaction ability. The core RAG1 contains all three catalytic residues of the DDE motif D600, D708, E962 that coordinate two divalent Mg^2+ ^ions essential for its activity, which represent the catalytic hallmark of most transposases and integrases an extended family to which RAG1 belongs.

Although RAG1 is the only subunit which specifically binds RSS DNA sequences, and contains all essential catalytic residues, RAG catalysis requires also the presence of RAG2. RAG2 a 527aa. protein by interacting with RAG1 enhances the complex DNA binding affinity, contains a plant homeodomain (PHD) finger (aa 384-527) that binds specifically to trimethylated histone H3 lysine 4 (H3K4me3), guiding RAG to regions of active chromatin^30^. RAG2 has no homology with any described transposase or viral integrase. Although RAG binds alone one 12-RSS forming the single or **s**ignal **c**omplex 12-SC, 23-SC and PC formation must be facilitated by an ubiquitous set of nuclear proteins HMGB1 or 2 which play an architectural role in bending the two 12/23-RSSs to fit into the final configuration compatible with catalysis^10,21,31,32^.

## 3. RAG1 similarities with other transposases

We will compare the RAG1 linear domain organization with that of other transposases used in this review as a reference for their DNA-protein complex conformations. Broadly speaking, we can assign to almost all DNA transposases and viral DNA integrases a trimodular functional character^27,33,34^. Each of these three types of modules may be mapped onto some small linear domains similarly positioned along these protein aa. sequences: 1. Specific DNA Binding Domain (DBD), 2. Catalytic Domain (CD) and 3. Nonspecific /Target DNA Binding Domain (TB).

### 3.1 Specific DNA Binding Domain (DBD)

All proteins from this family have a module which specifically binds the terminal IS (insertional) / IR (inverted repeat) DNA sequences of their mobile elements. They are domains folding independently and are located towards their N termini, (DBD shown in blue boxes in **Figure 1**). In some cases, these domains adopt well described motifs for other protein-DNA interactors. RAG1, 389-464 aa. NBD domain, adopts a symmetrical homodimer in which each monomer is bound to one nonamer element. Its N terminus recognizes the minor groove via an AT-hook-like GGRPR motif (R391 has base specific contacts with bottom T5, T6, whereas R393 with the phosphate of T5 of the nonamer) whereas the rest of the NBD adopts three α helices^11,35^. NBD Helix1 (400-407 aa.) is protruding deeply into the major groove with R402 contacting the conserved G2 bottom of the nonamer. Helix 2 and Helix 3 of one monomer form with those of the other NBD subunit a four-helix bundle involved in dimerization^35^. The NBD dimer presumably preserves the nonamer DNA orientation present in the PC.

**Figure 4:**
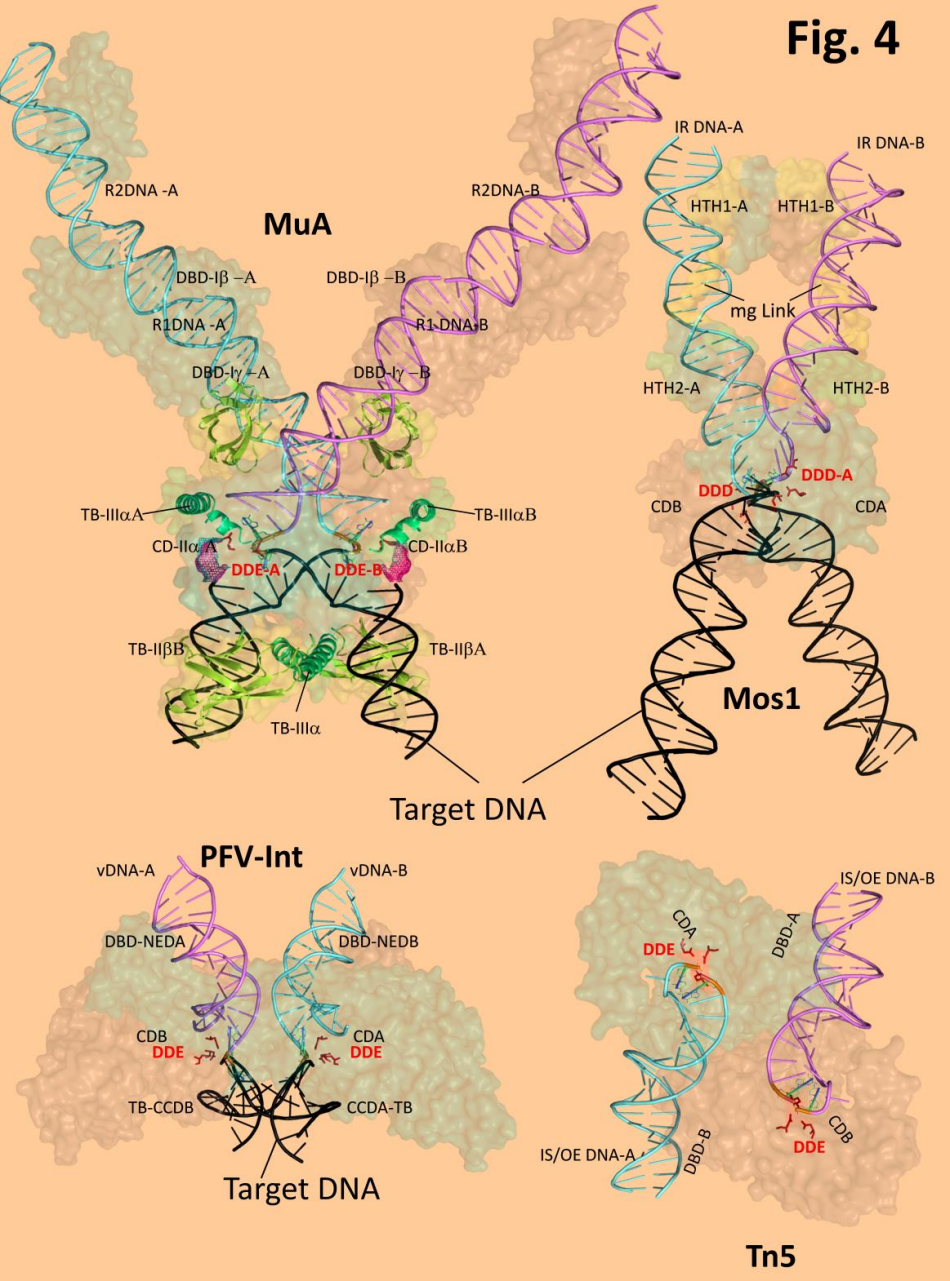
Diagram depicting 3D structures of MuA transpososome^16^(PDB code - 4CFY.pdb), Mos1PEC^18^ (PDB code - 3HOT.pdb), PFV-Intasome^15^ (PDB code - 3OS0.pdb) and of the Tn5 synaptosome^14^(PDB code - 1MUS.pdb), showing in color the positions of: the IS/IR DNA terminal sequences, DBD-DNA binding domains, CD-catalytic domains, DDE/D catalytic triad motif. in red, 3'OH and transferred DNA strand in orange, TB-target nonspecific DNA binding sites, with respect to each functional dual subunit (AB) organization of each complex.

Less is known about Hermes DBD (**Figure 1** [B]). Hermes transposase configuration has been studied in a hexameric multimer with a proposed three-fold dimer-subunit symmetry in which the N terminal 79-150 aa. domain involved in the terminal DNA element binding forms a composite inter-subunit 22 Å diameter channel^12^. MuA transposase (**Figure 1** [C], **Figure 4**) binds to the R or L terminal bacteriophage end via Iβ (76-168 aa.) and Iγ (178-242 aa.) domains using a winged helix DBD followed by a helix turn helix (HTH) domain the later a fold preserved by many members of the *mariner *family transposases^36,37^. Mos1 (**Figure 1** [D], **Figure 4**) from Tc1/mariner class recognizes the DNA IR using its 112 N terminal aa. grouped into two, triple α helical HTH motifs (HTH1, 8-53 aa. and HTH2, 74-110 aa.), connected by a linker that tightly binds in the minor groove, stabilizing the protein-DNA complex^18^.

Tn5 (**Figure 1** [E], **Figure 4**) uses its N terminal 70 aa. to bind specifically to its OE (outside end) IS elements using a compact four α helical bundle with the fourth one playing the "recognition helix" role since it protrudes in the major groove of the DNA bases 7 to 13 of the OE^14^. In this helix, an important K54 residue forms hydrogen bond with the O4 of thymine 10, and its mutation alters the DNA binding specificity of the transposase^14^.

### 3.2. The Catalytic Domain (CD)

The mid portion of these proteins contains the catalytic domains CD with the RNase H fold and their DDE/D catalytic motifs (in **Figure 1** shown in red boxes). For RAG1, this core domain containing the DDE motif was proposed to adopt the canonical RNaseH-like folding, or "retroviral integrase fold" with a three-layered α/β/γ domain centered by a five-stranded β sheet^26^. The exact fold (β1-β2-β3-α1-β4-α2/3-β5-α4-α5) shown in **Figure 2** has first been described in E. coli RNase H^38^ and then proved to be shared by structures of MuA^16,17^, Tn5^14^, Mos1^18^, Hermes^12^ transposases and by those of HIV-IN^39,40^ and PFV retroviral integrases. In these the first D is located on β1, the second on β4 and the third D/E is adjacent to α4. Based upon sequence folding prediction and domain organization homology RAG1 RNaseH-like folding was suggested by Dyda and coworkers to resemble that of the insect Hermes transposase^12,26^, with which RAG shares a common reaction mechanism (Figure 3and section 4). Given the long span between the first acidic D600, D708 pair of the motif and the glutamic acid in position 962, the authors propose that both transposases have an extended all α helical domain (aa. 761-979 in RAG1 and 265-300 in Hermes) inserted between their central core domain (residues 528-760 in RAG1 corresponding to 144-171 in Hermes) with the main integrase folding^12,26^, and both proteins C termini (**Figure 1** [A, B]). A particular distinct extended insertion domain was observed in the RNase H fold of the of Tn5 CD between the β5 and and α4, for this time the 96 stretch aa. is mostly made of a β-fold with a single α helix and four β-strands^14^.

In their work, Kapitonov and Jurka^9^ showed increased relative amino acids homology among sequences from diverse sources of RAG1 C-terminus CD with those from various transposases of *Transib s*uperfamily. The same work found a remarkable 35-38% identity between a 60-aminoacids C terminal portion of the Transib2 from the insect *Anopheles gambiae *transposase and the C-terminal portion of the core RAG1. Moreover, the sequence recognized by different Transib elements strikingly resemble those of RSSs, some having identical consensus heptamers and nonamers with those recognized by RAG but have spacers of various lengths. A recent report from Nancy Craig's group using an *in vitro* system with a purified Transib transposase *Hztransib* from the insect *Helicoverpa zea* showed that this enzyme has the same cleavage mechanism as RAG, forming a hairpin on the *Hztransib *DNA**end flank and a blunt double stranded cut on its signal end^41^ (see **Figure 3** [A]). The authors have added a mutational-functional analysis guided by sequence homology of *Hztransib *with the RAG1 CD and identified the enzyme to belong to a DDE type transposase, with its motif located as follows: D126, D225, E436. Unfortunately, little is known about the structure of these transposases to be able to draw more parallelism with our RAG1 enzyme, but these arguments strongly support the fact that RAG1 and Transib transposases might have a common ancestral progenitor.

### 3.3 Nonspecific/Target DNA Binding Domain (TB)

An interesting study from David Roth's group evidenced the fact that RAG proteins have considerably higher activity when cleaving RSSs located *in cis* (on the same DNA), if they were embedded on longer substrates^42^ than on short DNAs. The activity has been attributed to nonspecific RAG - DNA contacts which by 2D diffusion may facilitate the recruitment of RAG near the RSS. *In cis* versus *in trans* preference for 12/23-RSS synapsis was seen to be enhanced by negatively supercoiling the substrate DNA^43^, an effect which was similarly reported for many transposases and thought to be associated with target site binding preference^44^. Copper phenanthroline footprinting studies have shown that the last hundred aa. of RAG1 C terminus play a major role in nonspecific DNA binding via direct interactions with the coding flank DNA. Especially relevant was the description of K980A RAG1 mutant, which was defective in forming hairpin on long DNA^45^.

MuA transposase structure is perhaps the most relevant for TB topic, since its tetramer has extensive target DNA phosphate contacts mediated by IIβ and IIIα dmains (see **Figure 1**, **Figure 4**) all required for target DNA binding and bending. Especially revealing are the coiled-coil contacts of the dimerized IIIα domains located tightly on the concave side of the bent target DNA^16^.

In the case of the retroviral PFV integrase (**Figure 1** [F], **Figure 4**) its preference for YR (pyrimidine-purine) steps target integration is explained by the extended base contacts of R329 from its CTD (carboxy terminal domain) at the β1/β2 loop essential for target bending^15^ (**Figure 4**). Extended TB contacts are reported in the same study on PFV-IN between its CCD (catalytic core domain) α2 helix and both minor grooves of the target DNA (see **Figure 4**) via van der Waals interactions of the Cytosine 6, O2 and Ala 188 methyl group. Similar extended TB nonspecific DNA interactions have been reported for the C terminus domain of HIV integrase^46^ (**Figure 1** [G]).

## 4. RAG DNA cleavage mechanism; a comparative view with other transposase phosphoryl transfer reactions

**Figure 3** [A] (Inset) describes the RAG mechanism of cleavage at one RSS (shaded triangle). It occurs in two steps. First, an enzyme activated water molecule makes a nucleophilic attack on the scissile phosphate at the 5' coding / heptamer border, nicking the upper DNA strand. The free 3' hydroxyl group (3'OH) created at the 5' base of the heptamer attacks in the second step the phosphodiester bond of the bottom strand creating a hairpin (on the coding flank) and a blunt end DNA (on the signal end), thus cleaving the DNA. The events start with RAG-RSS SC binding, are followed by synapsis (PC) and end with the two catalytic cuts (SEC), a sequence that depicts the first phase of recombination. In a second phase, cleavage is followed by the joining of the coding ends, and is mediated by many of the general nonhomologous end joining/repair (NHEJ) machinery (XRCC4, Artemis, Ku70, Ku80, DNA-PKc, DNA Ligase IV) (see **Figure 3** left column mechanism RAG/ Hermes)^11,47^. Whereas the Ist strand nicking step can occur during a signal complex SC, hairpin formation requires the PC presence.

For an exhaustive analysis of the peculiarities of these reactions described in detail for various transposons the reader may consult the chapter written by K. Mizuuchi and T. Baker in Mobile Elements II book^48^. **Figure 3** schematically depicts the transposition/recombination reaction mechanisms of the main transposases with emphasis on four general steps: 1) Ist strand nicking which may occur at the final transferred DNA strand (Tn5, MuA) or alternatively on the nontransferred one (Hermes, RAG, Mos1), may occur prior to synapsis; 2) IInd strand nicking (Mos1) or hairpin formation on the signal end (Tn5) or on the opposite flank end (RAG, Hermes) creates double stranded breaks eliberating the transposon from its original donor. This step occurs only in the context of the synaptic complex (PC/PEC). 3) In RAG recombination reaction, the signal and the coding ends are separately ligated, generating the coding and signal joints, a process largely assisted by the ubiquitous set of cellular DNA repair enzymes; 4) Strand transfer reaction, in which the signal hairpin is opened (Tn5) and the attacking free 3'OH of the element terminal signals each is transferred to a phosphodiester bond of each of the two strands of the target. This reaction integrates the element into the host DNA. The attack creates a stagger between the two strands of the host chromosome which is duplicated at both ends of the element, after integration and DNA repair -target site duplication (TSD).

## 5. Paired complex organization and RSS bending in V(D)J recombination versus transpososome/ intasome architecture

Although the exact stoichiometry of RAG is still a subject of debate, many papers now convey that a functional recombinase is made of a dimer of RAG1 and two separate subunits of RAG2^29,49-51^. RAG heterotetramer has been described in 12 or 23-SC or in PC the two types of complexes assuming the same protein core^32,49,52^.

**Figure 5:**
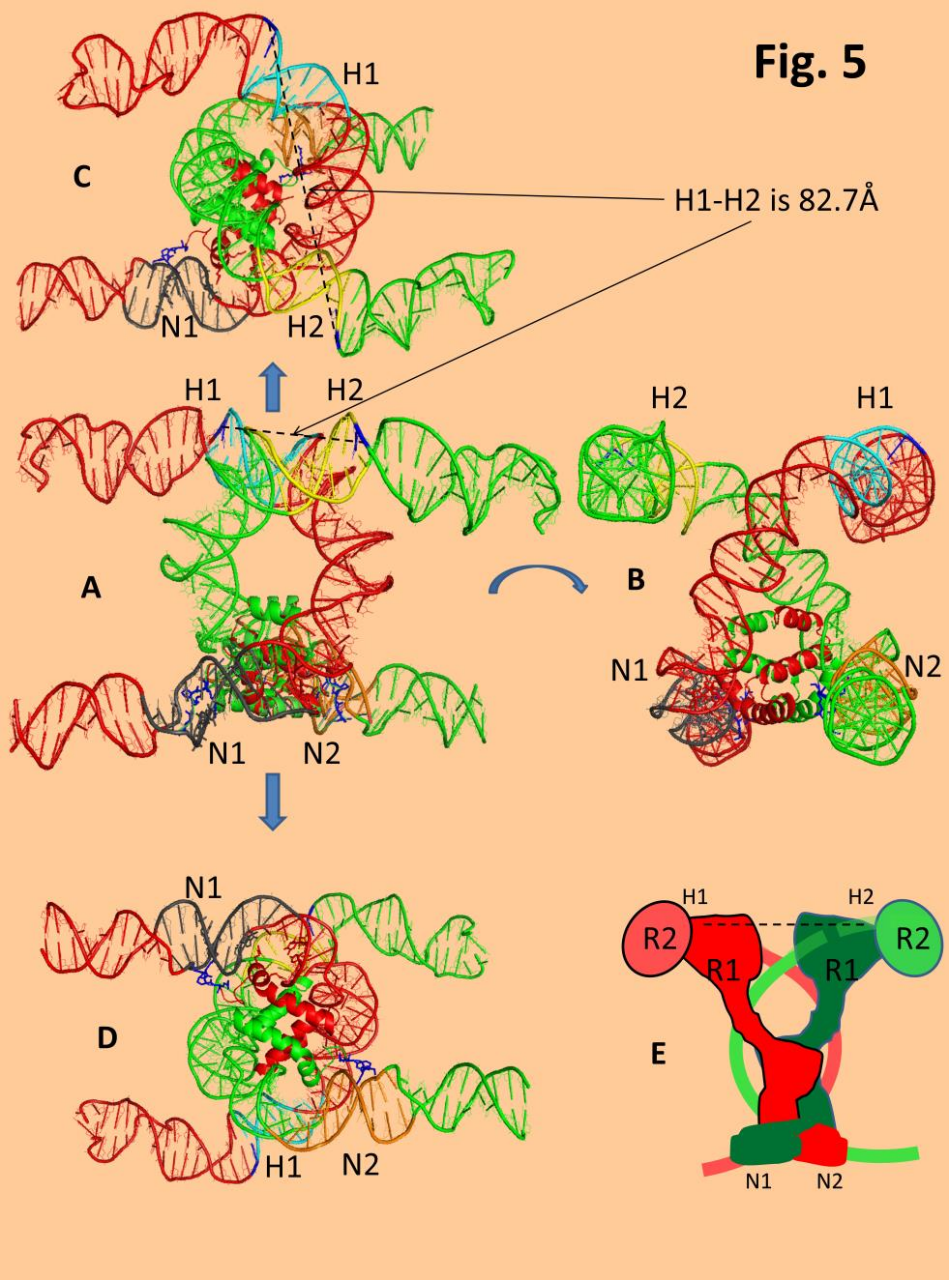
Model of the two bent 23-RSSs configured in the RAG PC, according to the FRET experimental constraints^55^. The model was realized by docking the dimeric NBD 389-464 aa. domain of RAG1 reported in5) (PDB code - 3GNA.pdb) onto the nonamer of the modeled PC bent 23-RSS. For docking the following constraints were respected: the O2 of the Thymidine [DT 79] and the O2 of Thymidine [DT 80] are contacted byArg391 NH2 side chain and the main Amide chain. The O6 of the guanine residue from the 23-RSS[DG 83] is also directly contacted by an Arg 402. In the 23-RSS, besides these Base-aa. direct contacts there are hydrogen bridges with the following phosphates: The phosphate of thymidine residue[DT 45] (with theNH2- of Lys405), and the phosphate of the cytidine [DC 44] (with the NH2- of Lys412). A- front view, B- side view 90 rotated, C-upper view , D-bottom view. The H1-H2 , measures the inter heptamer distance of the two 23-RSSs from the 5' phosphates of the 5' dC at heptamer coding flank junction(colored in navy blue). RSS1 in red has the Nonamer N1 colored in grey, and the heptamer H1 in light blue. Green RSS2 has the Nonamer colored in orange N2 and the heptamer H2 in yellow. E-cartoon representation of the best way to fit the SEC RAG protein organization modeled by Grundy et.al. 2009^50^, to our 2 RSS model. The peptidic AT-hook-like GGRPR motif of NBD is colored in navy blue.

To better understand transposases and RAG recombinase protein subunit organization one has to consider that synaptososome/ transpososome/ intasome or PC (or for some referred as PEC paired end complex) represents the key functional architecture of these enzymes, for this complex is the only one productive in terms of IInd strand nicking or hairpin formation (see **Figure 3**). The multimeric nature of all these enzymes define at least two functional subunits AB in which one right end DNA element whereas specifically engaged in interactions with the DBD of subunit A it is funneled towards the CD of the subunit B for catalysis, and the opposite rules the fate of the left end. **Figure 4** conveys this DNA-functional subunit organization reflected in four synaptososome structures: MuA, Mos1, Tn5 and PFV-IN. This *in trans* binding / catalysis tenet holds for all transposases and is a measure by which Nature avoids single uncorrelated DNA cuts which despite being sterile for the mobile elements can have dramatic effects for the host. Swanson's elegant in vitro RAG DNA binding/ cleaving study in which he assembled mixtures of wild-type with catalytically inactive RAG1 core heterodimers proved that RAG too adheres to this essential theme in which NBD and DDE motifs are located *in trans* on two RAG1-RAG2 functional subunits with respect to one RSS^51^. More recently Yang's and Gellert's groups have purified a cleaved 12/23 RSS - heterotetrameric (RAG1)_2 _(RAG2)_2_ complex SEC (signal end complex) and have studied its organization by electron negative staining and atomic force microscopy (AFM)^50^. The authors described the SEC highly symmetric model looking like an anchor in which the shank is made of two intertwined RAG1 monomers dimerized at the stem, then each diverging in opposite direction at the branching of the anchor's two arms. Each arm is ended at the drag of the anchor by a contact with one RAG2 monomer (see our cartoon representation of the model in **Figure 5** [E]). Although the AFM experiments of the study were aimed to position inside the complex the two heptamer processed RSSs, the technique is largely limited by the wide opaque protein profile which obstructs the view of the "inside complex" path of the DNA. Hence, one can only infer its entrance points of reference inside the complex, information that located the nonamers at the initial stem of the anchor. Similarly, AFM studies have been performed on RAG SC and PC with unprocessed RSSs of different length, but even in this case the "inside complex" trajectory of the DNA can only be inferred to accommodate large DNA bends^29,53^.

To study the organization of the DNA in the RAG PC, we used fluorescence resonance energy transfer FRET. In FRET measurements, the extent of non-radiative energy transfer efficiency E between two fluorescent dye molecules termed donor D and acceptor A can be calculated from the emission spectra of the two species, reporting the intervening distance when <100 Å. By FRET first, using 5' end uniquely labeled either with Donor (D) or Acceptor (A) fluorophore RSS oligonucleotides (their length ≈ 60bp ≈ 210 Å) we measured the end to end distances under conditions forming PC. All 8 combinations of the A/D fluorophore pairs located *in trans* (one on 12 and one on the 23-RSS) were compatible with resonance (interfluorophore distances < 80Å). A simple model with both RSSs contained in one plane and running side by side either in the same or opposite directions, as it was revealed for the Tn5/DNA structure (**Figure 4**)^14^, would be incompatible with our measurements. Instead, we favored a three-dimensional model where the two RSSs must be crossed and subjected to a considerable degree of bending which was measured for 23-RSS^54^. To elucidate the path of 23-RSS inside the PC we measured FRET with 23-RSS oligonucleotides internally labeled with fluorophores. We have revealed an intensely bent U shaped 23-RSS, with the arch located in the spacer whereas the heptamer and nonamer were present each on one arm of the letter^55^ (see **Figure 5**, each of the two 23-RSSs in the model is the result of our FRET study). Mapping the ethylation interference base contacts data of the 23-SC on our model we inferred the presence of RAG-HMGB1 contacts to be located at the concavity of the bent RSS.

In **Figure 4** we used the available high resolution transpososome/ intasome structural data available for Tn5^14^, MuA^16^, Mos1^18^ and PFV-IN^15,55,56^ and we compiled the data in order to convey a schematic two functional protein subunit AB (shown as colored semitransparent surfaces) with well defined highlighted DNA paths for each case. Our review aims to compare the DNA trajectories inside these complexes with that of the RSSs inside RAG PC. For those complexes where the target is present one can easily follow a X type configuration in which the two end DNA elements are brought convergently in an angle towards the "waist" of the X upper side of the structures (where the DBD of the proteins N termini are located). This narrow crossing contains the CDs, in which each DNA element is funneled with its 3'OH/ transferred strand onto the DDE/D motif of the other subunit than the one where its DBD contacts are. The only exception to this X scheme is that of the Tn5 structure where each OE is held in an antiparallel side by side planar orientation. Underneath the plane of the CDs that symmetrically contains the two DDE motifs is the arched profile of the target DNA surrounded by the adjacent TBDs. These TBDs together with the V of the target DNA build the lower arms of the letter X.

## 6. Recombination versus transposition: when the ends meet

There are several common structural themes from Mos1, MuA, Tn5 transpososomes and PFV Intasome (see **Figure 4**) which define a productive synaptosome prior to or during strand transfer to the target DNA. First, all of them have brought together the two reactive 3'OH groups from the IS/IR/LTR sequence elements after donor disintegration, each well suited in the proximity of one DDE motif of the catalytic subunits. The measured 3'OH-3'OH distance is for MuA ≈ 37.7 Å (is longer because it measures the distance post integration), for Mos1 ≈ 12.6 Å, for PFV-IN ≈ 21.6 Å and for Tn5 ≈ 44 Å. These 3'OH groups are symmetrically located for a nucleophilic attack that should occur in register each at one strand of the target phosphodiester that will define the borders of target site duplication. For MuA the target site duplication is of 5 bp^57^, for Mos1 is of 2 bp (dimer TA)^18^, for PFV Intasome is of 4 bp^15^ and for Tn5 is the longest of 9 bp^44^. Using a canonical B DNA with a helix rise of 3.4 Å between adjacent bases and a diameter of 20 Å we can roughly calculate the expected distance where the stagger of target site should occur. For MuA, the calculated distance between the attacked phosphodiesters would be of 26.25 Å, for Mos1 of 21Å, 24Å for PFV-IN and for Tn5 is of 30.6Å. RAG transposition creates a target site duplication of 5bp^4,7^, similar with that of MuA transposon insertion, and we assume roughly a similar order of magnitude for the distance between the two 3'OH of the heptamer groups in SEC, to allow strand transfer. Although there are clear discrepancies between these calculated distances and those measured from each structure, they do correlate with respect to their order of magnitude. Suffice to say that target DNA conformation revealed by these structures is far from that of the canonical B DNA. Instead, the target DNA is severely bent in MuA transpososome (140 bend)^16^ or PFV Intasome (125 bend)^15^. In Mos1 PEC although the authors have modeled a straight target B DNA, the actual crystal displays in each symmetry unit two duplex DNA oligonucleotides in a V shape arrangement right at the tip of the attacking IR DNA elements, mimicking again an extremely bent target^18^ (**Figure 4**). Bending a small rod shape DNA of lower length than the DNA persistence length is energetically unfavorable and requires either enthalpic support from protein binding or external constraints from the supercoiled state of the DNA. The extreme target DNA distortion is a "safety measure" requirement for at least two reasons. On one hand, the strand transfer reaction needs upon the synchronous double 3'OH attack on each of the two phosphodiesters flanking the target to quickly denature all adjacent hydrogen bonds from the intervening paired bases and the Van der Waals forces holding their stack in place by the intact strands. This is needed to dislodge the stagger and make room for the newly transferred transposon ends, an effect greatly facilitated by the bent shape of the target which weakens all these interactions. Secondly, once target integration occurred one has to prevent reversing the phosphoryl transfer reaction. This is efficiently achieved by pivoting the base at the IS/IR target junction (near the tip of the target bend), which moves the DDE- Mg^2+^ from its original catalytic center, an effect nicely portrayed by MuA and PFV-Int structures^15,16^. Again, such base pivoting is enhanced by the weak stacking at the tip of the sharp DNA bends. But whereas target DNA bending seems to be advantageous in helping strand transfer in transposition and viral DNA integration, what kind of consequences one expects to happen if such large bends are induced into the IS/IR/RSSs prior to the target invasion, in the synaptosome (PC)? This seems to be the situation with RAG-RSS in PC and to some extent at nonamer level in SEC. Trying to answer this question, we docked the structure of the RAG1 NBD 389-464 aa. dimer^35^ onto the nonamer of our modeled PC bent 23-RSS^55^ (**Figure 5**), keeping the major constraints of the base-Aa contacts as described in **Figure 5** legend. The NBD dimer interface of the structure guided us in orienting the second RSS which we artificially assigned to be another PC bent 23-RSS, identical with the one bound to the first NBD monomer, but now responding to the nonamer base-Aa. binding constraints of the second monomer from the NBD dimer structure. This model has serious limitations coming from the pairing of two 23-RSSs which is being mediated just by a small 75 aa. polypeptide from the total of 1040 aa. of the full length RAG1. However, for the sake of the raised argument we will just consider its orientation and its overall dimensional value, neglecting any detailed contacts or exact residue positioning that may be implied by the model. Moreover, our work in progress shows that 12-RSS adopts in RAG, HMGB1, PC and SC a very similar bent configuration (Ciubotaru M et al. unpublished, and data not shown) with the one documented in our last study on 23-RSS in the PC^55^. **Figure 5** depicts several views of this model which can be schematized by three major traits: 1) Each of the two bent RSSs has a horizontal U shape with their arches intertwined in the center and the four arms pointing outwards to the left and to the right (**Figure 5** [A]); 2) The upper two arms of the letters are occupied by the heptamers (**Figure 5** [C] top view) whereas the bottom arms by the two nonamers (**Figure 5** [D] lower view ); 3) **Figure 5** [B] shows that in a side view incidence each of the planes of the two U shaped RSSs is tilted making a 75 degree with the horizontal, the letter planes crossing in an uneven X projection with the wider lower arms accommodating in between them the NBD. The projection of our model was rebuilt on the organization of the (RAG1)_2 _(RAG2)_2_ SEC revealed by Grundy et. al.^50^ and is shown in **Figure 5** [E]. We point here that our two RSS model in this projection is consistent with the transpososome assembly tenet according to which one RAG1-RAG2 subunit binds the nonamer of one RSS whereas the other adjacent subunit would catalize its heptamer *in trans*^51^.

If one relates this model image to those of MuA transpososome, Mos1 PEC or of the PFV-Intasome, one comes to appreciate that RAG PC is in fact a version that offers direct continuity between each arm of one of the DNA transposon end/RSS nonamer and one arm of the arched target DNA/ RSS heptamer. It is as if one has flipped the plane of the two transpososome CDs upside down and now the two DDE motifs of each of the two CDs instead of pointing inwards convergently towards the center of the transpososome point outwards divergently at the top of the PC. If this observation is correct it would only reveal that RAG could have evolved towards recombination by plying apart the two DDE motifs in its SEC/PC. This would in fact separate the two signal RSS 3'OH groups too far apart for their concerted attack to occur on a target DNA in order to create a convenient stagger. To test this bold hypothesis, we needed to approximate the distance between the two 3'OH of the cutted RSS signals in the SEC, or at least to estimate the distance between the two nearby DDE motifs. Given that no high resolution structural information is available for the RAG CD we do not have an exact way to position each RAG CD subunit DDE motif on our paired RSSs model. However, UV crosslinking studies have revealed the first 5' dC present at heptamer /coding junction points towards the RAG1-RAG2 catalytic surface^58^. This residue was found crosslinked to both RAG proteins at their interface. We have labeled with navy blue the position of this dC where first strand nicking initiates the cleavage, and measured the phosphate-phosphate distance between these residues on the two modeled RSSs to be of 82.7Å (**Figure 5** [A], D H1-H2 distance). Given the limitations of our model, although we cannot put too much emphasis on the exact value of this measurement, its order of magnitude certainly supports our assumptions. A distance longer than that of two B DNA helices (70Å) between the two attacking 3'OH groups would definitely be incompatible with any strand transfer target stagger, no matter how efficiently this DNA would be bent to denature the intervening staked bases. Nonetheless, if RAG evolution has changed the functionality of these proteins to carry out recombination instead of transposition how can we explain then the fact that using core 384-1008 RAG1 and core RAG2 1-387 truncated proteins instead of full length counterparts, one can generate RAG mediated transposition with a target site duplication of 5bp? The answer seems to be hinted by RAG's closest relative Hermes. Hermes is expressed in soluble form as a truncated N terminus version 79-612 and shown to be active only when present as a hexameric multimer^12^. In their study, Dyda and coworkers elegantly showed that the multimeric nature of this protein helps juxtaposing *in trans* two DDE motifs in close vicinity (40Å) from two adjacent dimers, thus creating a symmetrical CD interface compatible with a 8 bp stagger (≈ 30 Å). A similar effect may be induced if core RAG proteins would oligomerize > heterotetramer generating *in trans* a dual proximal juxtaposition of two DDE motifs, coming from distinct tetramers at a multimerization interface that may be compatible with a 5 bp target stagger. Recent AFM studies showed that Ca^2+^ and low ionic strength buffers induce RAG octamers which were associated with RSSs^29,53^. Suffice to say that in vitro transposition assays are usually carried out by first assembling the RAG SEC in Ca^2+^ which blocks RAG activity and then the SEC is resuspended in buffers with Mg^2+^ and the target DNA, thus perhaps enabling RAG multimerization^5,7^.

## 7. Conclusions

We will conclude our review underscoring the striking structural and functional similarities among RAG1 and some of the best understood transposases and we propose based upon structural evidence from our work, an evolutionistic hypothesis that could have changed the outcome of an ancient transposition reaction into that of V(D)J recombination.

- **In all jawed vertebrates V(D)J recombination performed by RAG recombinase is the essential process creating their specific immunity. Although the recombinase can inefficiently transpose intersignal DNA, and RAG1 resembles cut and paste transposases, evolution has changed RAG to perform mainly recombination during lymphocytes development. **

- ***How evolution has changed RAG's profile and reaction mechanism*** ***from that of a transposase into that of a recombinase?*** **In this review we offer some clues coming from the structural analysis of how DNA is bent and organized in various transpososomes versus how the DNA is organized inside the recombinase PC, that may explain this essential conundrum.**
